# Potential Protective Role of Galectin‐3 in Airway Dilatation in Obstructive Airway Diseases

**DOI:** 10.1002/clt2.70092

**Published:** 2025-08-06

**Authors:** Mariko Kogo, Hisako Matsumoto, Naoya Tanabe, Chie Morimoto, Natsuko Nomura, Hironobu Sunadome, Tadao Nagasaki, Susumu Sato, Atsuyasu Sato, Tsuyoshi Oguma, Isao Ito, Toyohiro Hirai

**Affiliations:** ^1^ Department of Respiratory Medicine Graduate School of Medicine Kyoto University Kyoto Japan; ^2^ Department of Respiratory Medicine and Allergology Kindai University Faculty of Medicine Osaka Japan; ^3^ Department of Respiratory Care and Sleep Control Medicine Graduate School of Medicine Kyoto University Kyoto Japan; ^4^ Department of Respiratory Medicine Kindai University Nara Hospital Osaka Japan

To the Editor,

Concomitant bronchiectasis is receiving increasing attention in airway diseases including asthma and chronic obstructive pulmonary disease (COPD). Studies have described the association between concomitant bronchiectasis and poor outcome in these diseases [1, 2]. Although the role of neutrophilic inflammation in the concomitant bronchiectasis in asthma and COPD has been suggested [3], the potential role of efferocytosis, the process by which phagocytes remove apoptotic host cells, remains unclear.

Efferocytosis is known to be impaired in severe asthma, particularly in neutrophilic asthma [4], and COPD [5]. Impaired efferocytosis can lead to persistent inflammation through the accumulation of dead cells, airway destruction and the development of bronchiectasis. Galectin‐3, a β‐galactoside‐binding lectin, is highly expressed in myeloid cells including macrophages, fibroblasts, epithelial cells, and endothelial cells, and extracellular galectin‐3 enhances efferocytosis of macrophages [5, 6]. Gao et al. described that sputum galectin‐3 is reduced in neutrophilic asthma compared with eosinophilic asthma [4]. However, its role in airway dilatation in obstructive airway diseases remains unknown.

This study aimed to investigate the role of galectin‐3 in airway dilatation in patients with obstructive airway diseases.

We used a closed cross‐sectional study that prospectively enrolled stable COPD and asthma patients with airflow obstruction (forced expiratory volume in one second [FEV_1_]/forced vital capacity [FVC] < 0.7) [3]. The details regarding the diagnosis are presented in Supporting Information S1: Methods and Table S2. Patients were enrolled between 2019 and 2020 at Kyoto University Hospital and sputum samples were collected at least 4 weeks after exacerbation resolution. Among 68 participants, 43 with available computed tomography (CT) scans and sputum samples were analyzed for cell differentiation, microbiome analysis and galectin‐3 measurement (R&D Systems, DGAL30). Inflammatory phenotypes were classified based on sputum cell differentiation as follows: neutrophilic phenotype: neutrophils ≥ 60%, eosinophilic: neutrophils < 60% and eosinophils ≥ 2%, and pauci‐granulocytic: neutrophils < 60% and eosinophils < 2%. The high galectin‐3 group comprised patients in the upper tertile of galectin‐3 levels (≥ 217 ng/mL). Airway dilatation was defined as the internal diameter of the bronchus being greater than that of the adjacent pulmonary artery or the absence of a tapering of the bronchial lumen towards the periphery in any generation on CT [3].

Among 43 patients analyzed, 11 patients presented airway dilatation on CT at enrollment. Patients with airway dilatation had a higher proportion of γ‐*Proteobacterium* in sputum, a higher COPD assessment test score, and a trend towards a lower prevalence of the eosinophilic phenotype than those without (Table 1). The prevalence of patients with high galectin‐3 (≥ 217 ng/mL) tended to be lower in patients with airway dilatation than those without (*p* = 0.06). Notably, no patient with high galectin‐3 presented airway dilatation in neutrophilic phenotype (Figure 1). The subgroup analysis by diagnosis is presented in Figure S1. In eosinophilic phenotype, galectin‐3 levels were unrelated to airway dilatation. To confirm the results, an additional analysis was performed on patients with FEV_1_/FVC < lower limits of normal [7] (*n* = 41), which provided similar findings (Figure S2).

**TABLE 1:** Characteristics of patients, stratified by the presence of airway dilatation.

	With airway dilatation (*n* = 11)	Without airway dilatation (*n* = 32)	*p* value
Age, y	72 (63–78)	72 (67–76)	0.89
Male, *n* (%)	6 (55)	27 (84)	0.043
Body mass index, kg/m^2^	23 (21–28)	23 (21–25)	0.68
≥ 10 pack/year, *n* (%)	8 (73)	22 (69)	0.80
Diagnosis			0.28
Asthma	0 (0)	6 (19)	
COPD	2 (18)	6 (19)	
Asthma and COPD overlap	9 (82)	20 (63)	
Age of onset (or diagnosis), y	49 (30–55)	55 (39–63)	0.30
%Predicted FEV_1_, %	71 (47–90)	76 (62–86)	0.54
FEV_1_ < 50% predicted, *n* (%)	4 (36)	4 (13)	0.17
FEV_1 /_ FVC < LLN, *n* (%)	10 (91)	31 (97)	0.45
FeNO, ppb	35 (15–48)	39 (24–66)	0.24
Blood eosinophil count,/μL	169 (71–608)	358 (164–550)	0.28
ICS Use, *n* (%)	10 (91)	27 (84)	1.00
Macrolide use, *n* (%)	2 (18)	4 (13)	0.64
OCS Use, *n* (%)	2 (18)	6 (19)	1.00
Exacerbation within 1 year prior to inclusion	3 (27)	2 (7)	0.11
Sputum inflammatory phenotype			0.14
Pauci‐granulocytic	1 (9)	0 (0)
Eosinophilic	1 (9)	8 (25)	
Neutrophilic	9 (82)	24 (75)	
Class γ *Proteobacterium*, %	11 (5–30)	5 (3–7)	0.01
Genus *Haemophilus*, %	7 (2–11)	3 (1–6)	0.053
Genus *Streptococcus*, %	11 (7–17)	15 (11–19)	0.17
Genus *Porphyromonas*, %	2 (0.04–3)	2 (1–5)	0.35

*Note:* Values indicate the median (interquartile range). *p* values were calculated using chi‐squared test, Fisher's exact test or Wilcoxon rank sum test, where appropriate.

Abbreviations: COPD, chronic obstructive pulmonary disease; FeNO, fractional exhaled nitric oxide; FEV_1_, forced expiratory volume in one second; FVC, forced vital capacity; ICS, inhaled corticosteroid; LLN, lower limits of normal; OCS, oral corticosteroid.

**FIGURE 1:**
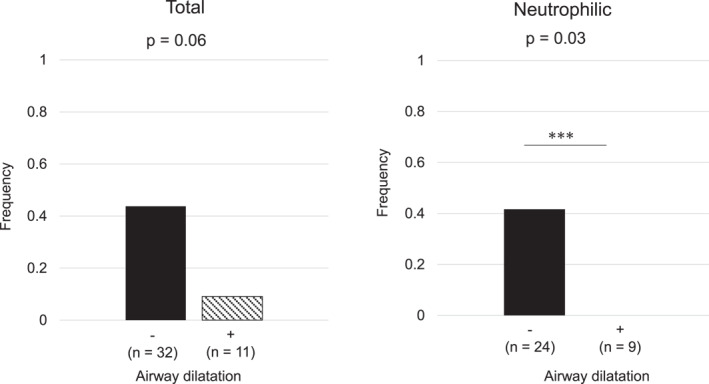
The frequency of patients with high galectin‐3 among patients with and without airway dilatation according to sputum inflammatory phenotype.

Secondly, we evaluated the association between galectin‐3 and inflammatory cells in sputum. Overall, galectin‐3 exhibited a negative correlation with sputum neutrophils (rho = −0.31, *p* = 0.046). Although the association with eosinophils was not significant (rho = 0.19, *p* = 0.22), the prevalence of patients with high galectin‐3 increased numerically in patients with severe airway eosinophilia (25%, 25%, and 53% in the low [< 2%], medium [2%–8%], and high [> 8%] eosinophil groups, respectively, *p* = 0.10).

In the neutrophilic group (*n* = 33), galectin‐3 positively correlated with macrophage count (rho = 0.47, *p* < 0.01), but not with neutrophil or eosinophil count (rho = −0.23, *p* = 0.21 and rho = 0.01, *p* = 0.98, respectively). Meanwhile, in the eosinophilic group (*n* = 9), galectin‐3 strongly correlated with sputum eosinophil count (rho = 0.82, *p* < 0.01).

This study demonstrated that low galectin‐3 levels in sputum were associated with the presence of airway dilatation, particularly in the neutrophilic obstructive airway diseases. In addition to the known negative correlation between sputum galectin‐3 levels and neutrophil count [4], in this study galectin‐3 was associated with macrophages in the neutrophilic group.

Impaired clearance of apoptotic cells in cystic fibrosis and bronchiectasis has been reported previously [8]. Our study suggests that a similar process may occur in the development of concomitant bronchiectasis in COPD and asthma. Reduced galectin‐3 may impair efferocytosis [5] and cause airway destruction, via reactive oxygen species or proteases from apoptotic neutrophils. Although we did not directly assess efferocytosis, this is one of the first studies to investigate the mechanism of development of bronchiectasis in obstructive airway disease from the perspective of efferocytosis.

In the eosinophilic phenotype, sputum galectin‐3 levels were associated with eosinophil counts, which is consistent with a previous finding that galectin‐3 facilitates eosinophil rolling and adhesion [9]. Additionally, given the antibacterial property of galectin‐3 [6], elevated galectin‐3 may indirectly contribute to the reduced risk of bacterial infection in the eosinophilic phenotype.

This study has several limitations, including a small sample size, which prevents firm conclusions and limits the ability to perform detailed comparisons between asthma, COPD, and their overlap. Nevertheless, this study suggests that galectin‐3 may play different roles in asthma and COPD: it may enhance eosinophilic airways in typical eosinophilic asthma, but may prevent the development of bronchiectasis in neutrophilic airways, a hallmark of COPD.

In conclusion, our findings provide important insights into the potential protective role of galectin‐3 in the development of bronchiectasis in neutrophilic obstructive airway diseases, although large‐scale longitudinal studies are required.

## Author Contributions

**Mariko Kogo:** conceptualization, methodology, writing – original draft, investigation, data curation, visualization. **Hisako Matsumoto:** project administration, conceptualization, methodology, investigation, resources, writing – review and editing, data curation, funding acquisition, visualization. **Naoya Tanabe:** project administration, writing – review and editing, supervision, data curation, investigation, visualization. **Chie Morimoto:** project administration, data curation, methodology, writing – review and editing, investigation. **Natsuko Nomura:** investigation, data curation, writing – review and editing. **Hironobu Sunadome:** writing – review and editing, data curation, investigation, project administration. **Tadao Nagasaki:** data curation, writing – review and editing. **Susumu Sato:** data curation, supervision, writing – review and editing. **Atsuyasu Sato:** writing – review and editing, data curation. **Tsuyoshi Oguma:** data curation, writing – review and editing. **Isao Ito:** data curation, writing – review and editing. **Toyohiro Hirai:** supervision, conceptualization, writing – review and editing

## Conflicts of Interest

The authors declare no conflicts of interest.

## Supporting information

Supporting Information S1

Figure S1

Figure S2

## Data Availability

The data that support the findings of this study are available upon request from the corresponding author. The data are not publicly available due to privacy or ethical restrictions.
