# Supplementary material for: Potential Protective Role of Galectin‐3 in Airway Dilatation in Obstructive Airway Diseases
Source: Clin Transl Allergy. 2025 Aug 6;15(8):e70092. doi: 10.1002/clt2.70092 (PMC12328036; doi:10.1002/clt2.70092)
Supplement: Supplementary file 1 — Supporting Information S1 [file CLT2-15-e70092-s001.docx]

**Potential protective role of galectin-3 in airway dilatation in obstructive airway diseases**

**Unmarked Supporting information**

**Methods**

Asthma and COPD were diagnosed based on the Global Initiative for Asthma^1^ and the Global Initiative for Chronic Obstructive Lung Disease reports^2^. ACO was subsequently identified based on the management guideline for ACO (Table S2) published by the Japanese Respiratory Society^3, 4^.

**Supplementary References**

1. Reddel HK, Bacharier LB, Bateman ED, et al. Global Initiative for Asthma Strategy 2021: Executive Summary and Rationale for Key Changes. Am J Respir Crit Care Med 2022: 205: 17-35.
2. Agusti A, Celli BR, Criner GJ, et al. Global Initiative for Chronic Obstructive Lung Disease 2023 Report: GOLD Executive Summary. Am J Respir Crit Care Med 2023: 207: 819-837.
3. Hashimoto S, Sorimachi R, Jinnai T, Ichinose M. Asthma and Chronic Obstructive Pulmonary Disease Overlap According to the Japanese Respiratory Society Diagnostic Criteria: The Prospective, Observational ACO Japan Cohort Study. Adv Ther 2021; 38 1168-1184.
4. Morimoto C, Matsumoto H, Nomura N, et al. Sputum microbiota and inflammatory subtypes in asthma, COPD, and its overlap. J Allergy Clin Immunol Glob 2024;3(1):100194.

**Table S1. Characteristics of patients, stratified by asthma, COPD, and their overlap**

|  | Asthma  (n =6) | COPD  (n = 8) | Overlap  (n = 29) | *P* value |
| --- | --- | --- | --- | --- |
| Age, y | 74 (68 – 83) | 73 (71 – 81) | 71 (64 – 74) | 0.18 |
| Male, n (%) | 3 (50) | 7 (88) | 23 (79) | 0.27 |
| Body mass index, kg/m^2^ | 22 (20 – 24) | 24 (22 – 27) | 23 (21 – 25) | 0.38 |
| ≥ 10 pack/year, n (%) | 0 (0) | 7 (88) | 23 (79) | < 0.01 |
| Age of onset (or diagnosis) | 43 (27 – 65) | 60 (52 – 67) | 53 (34 – 58) | 0.08 |
| %predicted FEV_1_, %  FEV_1_ <50% predicted, n (%) | 86 (63 – 102)  1 (17) | 79 (63 – 89)  1 (13) | 71 (52 – 81)  6 (21) | 0.25  1.00 |
| FEV_1 /_ FVC < LLN, n (%) | 5 (83) | 7 (88) | 29 (100) | 0.10 |
| FeNO, ppb | 55 (42 – 77) | 25 (13 – 30) | 41 (25 – 79) | 0.01 |
| Blood eosinophil count, /μL | 414 (186 – 1040) | 244 (83 – 305) | 334 (111 – 604) | 0.44 |
| ICS use, n (%) | 6 (100) | 3 (38) | 28 (97) | < 0.01 |
| Macrolide use, n (%) | 0 (0) | 0 (0) | 6 (21) | 0.72 |
| OCS use, n (%) | 1 (17) | 0 (0) | 7 (24) | 0.36 |
| Exacerbation within 1 year prior to inclusion | 0 (0) | 1 (13) | 4 (14) | 1.00 |
| Sputum inflammatory phenotype  Pauci-granulocytic  Eosinophilic  Neutrophilic | 0 (0)  2 (33)  4 (67) | 0 (0)  0 (0)  8 (100) | 1 (3)  7 (24)  21 (72) | 0.46 |
| Class γ *Proteobacterium*, % | 2 (1 – 7) | 6 (1 – 13) | 7 (4 – 11) | 0.09 |
| Genus *Haemophilus*, % | 0.9 (0.4 – 4.2) | 3.7 (1.3 – 7.9) | 4.3 (1.7 – 7.2) | 0.15 |
| Genus *Streptococcus*, % | 14 (11 – 30) | 12 (9 – 16) | 15 (11 – 19) | 0.37 |
| Genus *Porphyromonas*, % | 1.7 (0.6 – 4.2) | 5.6 (0.9 – 7.0) | 1.9 (0.3 – 3.5) | 0.11 |

Values indicate the median (interquartile range). P values were calculated using Chi-squared test, Fisher’s exact test or Wilcoxon rank sum test, where appropriate. COPD, chronic obstructive pulmonary disease. FeNO, fractional exhaled nitric oxide. FEV_1_, forced expiratory volume in one second. FVC, forced vital capacity. ICS, inhaled corticosteroid. LLN, lower limits of normal. OCS, oral corticosteroid.

**Table S2. Japanese Respiratory Society diagnostic criteria for asthma and COPD overlap^3^**

| Basic criteria | |
| --- | --- |
| Age ≥ 40 years and chronic airflow obstruction: post-bronchodilator FEV_1_/FVC < 70% | |
| [Characteristics of COPD]  One item from 1, 2, and 3 | [Characteristics of asthma]  Two items from 1, 2, and 3, or  One item from 1, 2, and 3 and at least two items from 4 |
| 1. Smoking history (10 pack-years or more) or career involving significant air pollution or biomass exposure  2. Presence of low attenuation areas on chest CT demonstrating emphysematous changes  3. Impaired pulmonary diffusing capacity (%*D*_LCO_ < 80% or %*D*_LCO_/*V*_A_ < 80%) | 1. Variable (diurnally, daily, and seasonally) or paroxysmal respiratory symptoms (cough, sputum, and dyspnea)  2. History of asthma before age 40 years  3. FeNO > 35 ppb  4. (1) Concomitant perennial allergic rhinitis  (2) Airway reversibility (change in FEV_1_ > 12% and > 200 mL)  (3) Peripheral blood eosinophils > 5% or > 300/μL  (4) High IgE level (total IgE, or IgE specific to perennial inhalant antigens) |
| 1. To be diagnosed as ACO, one item of the characteristics of COPD plus two items from 1, 2, and 3 or one item from 1, 2, and 3 and at least two items from criterion 4 of the characteristics of asthma are needed  2. If the characteristics of COPD alone are present, it is diagnosed as COPD, and if the characteristics of asthma alone are present, it is diagnosed as asthma (with remodeling)  3. If the characteristics of asthma cannot be confirmed when diagnosing ACO, it is important to monitor for the presence of the characteristics of asthma over time  4. Perennial inhalant antigens include house dust, mites, molds, scales from animals, and feathers, and seasonal inhalant antigens include pollen from trees, plants, and weeds  Note 1. Diseases of differential diagnosis (diffuse panbronchiolitis, congenital sinobronchial syndrome, obstructive panbronchiolitis, bronchiectasis, pulmonary tuberculosis, pneumoconiosis, lymphangioleiomyomatosis, congestive heart failure, interstitial lung disease, and lung cancer) should be ruled out by standard chest x-rays, etc.  Note 2. Respiratory symptoms such as cough, sputum, and dyspnea are variable (diurnally, daily, and seasonally) or paroxysmal in asthma and chronic and continuous in COPD | |

COPD, chronic obstructive pulmonary disease. CT, computed tomography. D_LCO,_ diffusion capacity for carbon monoxide. FeNO, fractional exhaled nitric oxide. FEV_1_, forced expiratory volume in one second. FVC, forced vital capacity. Ig, immunoglobulin. V_A_, alveolar volume.

**Figure S1.** The frequency of patients with high galectin-3 levels among those with and without airway dilatation in COPD (n = 8) and ACO (n = 29). No patients with asthma showed airway dilatation. All patients diagnosed with COPD had neutrophilic inflammation.

**Figure S2.** The frequency of patients with high galectin-3 levels among those with and without airway dilatation, according to sputum inflammatory phenotype (with airflow obstruction defined as FEV_1_/FVC < lower limits of normal).
