# Supplementary figures and images for: Potential Protective Role of Galectin‐3 in Airway Dilatation in Obstructive Airway Diseases
Source: Clin Transl Allergy. 2025 Aug 6;15(8):e70092. doi: 10.1002/clt2.70092 (PMC12328036; doi:10.1002/clt2.70092)

Figure S1

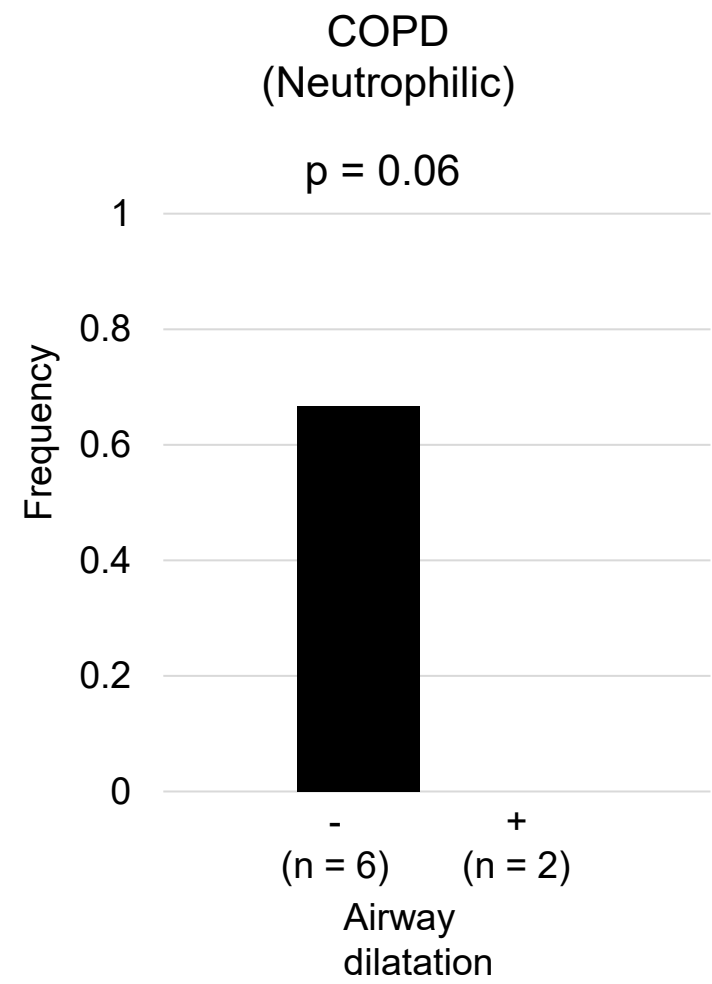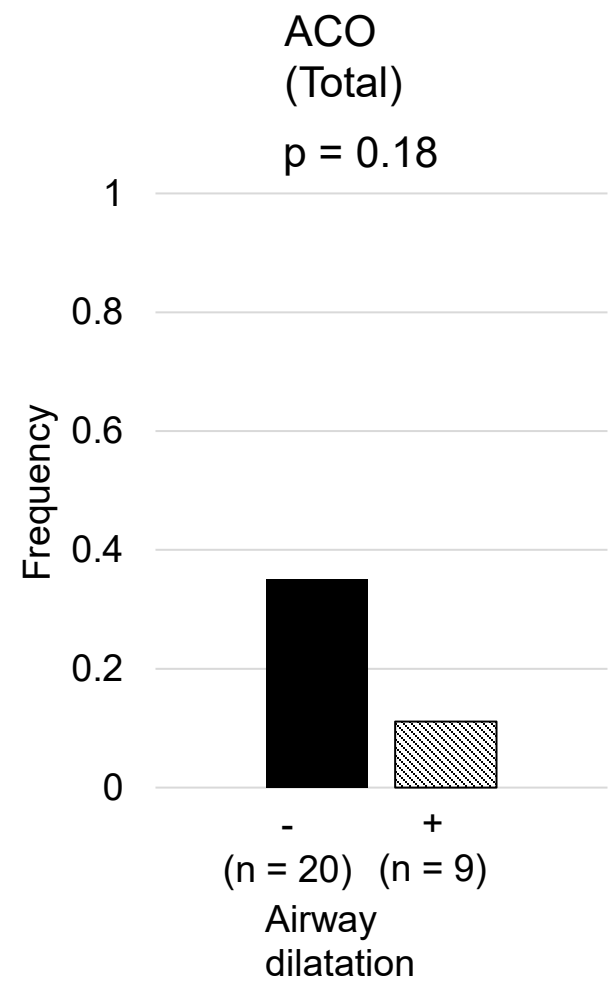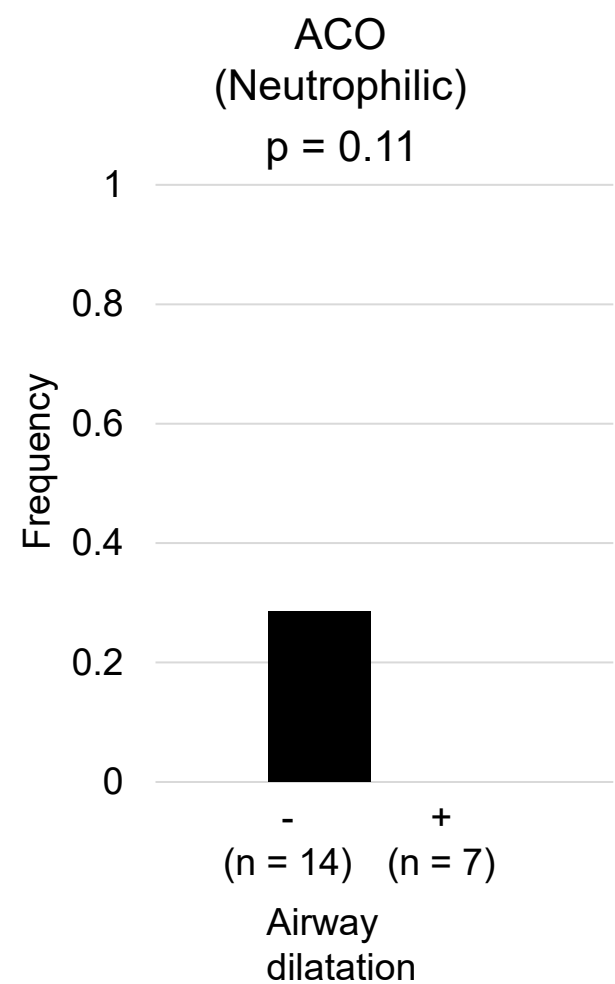

Supplement: Supplementary file 2 — Figure S1 [file CLT2-15-e70092-s002.pdf]

Figure S2

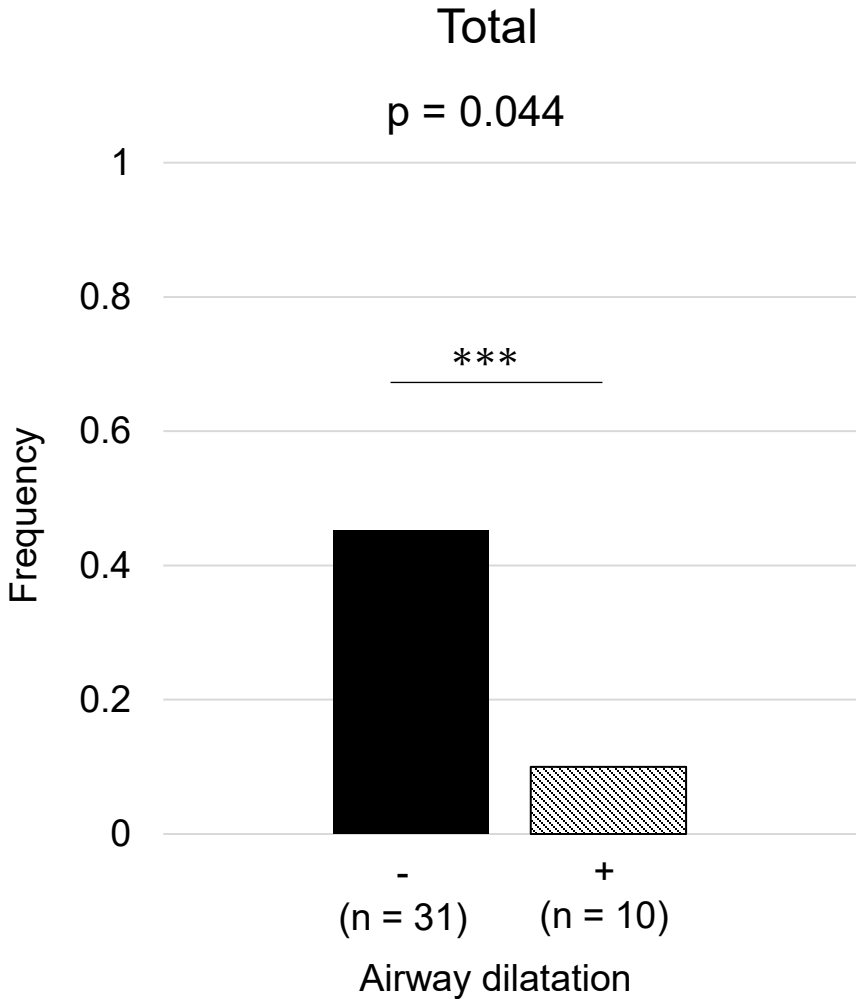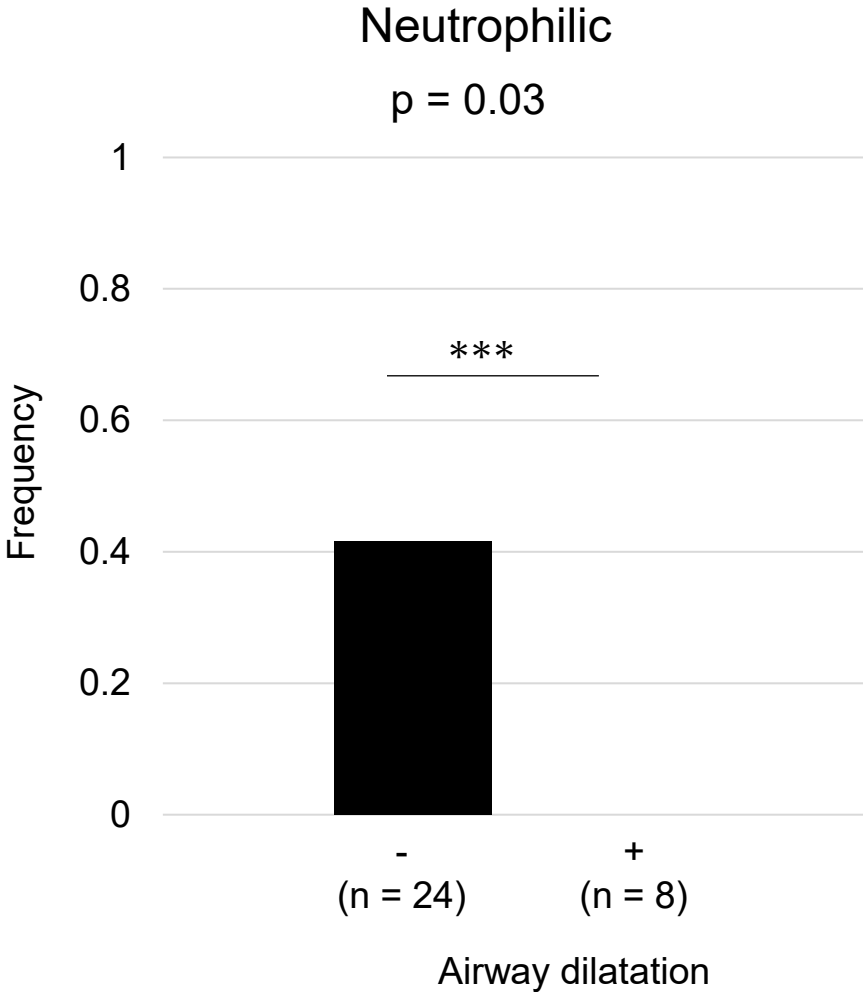

Supplement: Supplementary file 3 — Figure S2 [file CLT2-15-e70092-s003.pdf]
